# The Proposed State Board of Health

**Published:** 1902-09

**Authors:** 


### TEXAS MEDICAL JOURNAL,

AUSTIN, TEXAS.

A MONTHLY JOURNAL OF MEDICINE AND SURGERY.

EDITED AND PUBLISHED BY
F. E. DANIEL, M. D.

ASSOCIATE EDITOR:
WITTEN BOOTH RUSS, M. D.,
San Antonio, Texas.

Published Monthly at Austin, Texas. Subscription price $1.00 a year in advance.

Eastern Representative: John Guy Monihan, St. Paul Building, 220 Broadway, New York City.

Official organ of the the West Texas Medical Association, the Houston District Medical Association, the Austin District Medical Society, the Brazos Valley Medical Association, the Galveston County Medical Society, and several others.

### THE PROPOSED STATE BOARD OF HEALTH.

At the request of the State Medical Association's Committee on a State Board of Health, President Red has added Dr. Geo. R. Tabor, State Health Officer, to the committee. He is thoroughly in accord with the committee as to the construction of such a board as is contemplated, and will co-operate with them in the effort to secure legislation enlarging the scope and powers of the health department. It is by no means contemplated by the committee to ask for the abolition of the Quarantine Department, or to materially interfere with its operation, but rather to build on it, as a foundation, a Public Health Department, which shall embrace quarantine as its chief feature, and perhaps ask to change the name from Quarantine Department as it now stands to Public Health Department, making the State Health Officer (Quarantine Officer) ex-officio President of the State Board of Health, and entrusting him as now with the quarantine management. The aim will be to so enlarge the powers and authority of the department as to collect, preserve and publish the vital statistics of the State, and to supervise and control local as well as general sanitation, enforcing measures of prevention against diseases of local origin as well as those of foreign origin, and the spread of contagious diseases. It is contemplated to create the office of Secretary of the Board who shall, in addition to the duties devolving upon that office, be the Registrar of the Vital Statistics; a chemist, who shall be, also, the bacteriologist and pathologist of the Board, and two or three sanitary advisors in different parts of the State who shall have the sanitary supervision of their respective districts, and who shall meet with the Board and consult, when consultation is necessary, as to quarantine and other matters. It is also contemplated to have a local board of health in every county and in every incorporated town, and also a health officer of every county, to be paid by the county, and who shall be subordinate to the State Board and a part of the machinery of the Health Department of the State. The Board must make and publish a sanitary code for the State and have power to enforce, under penalties, its mandates for the protection and preservation of the public health. Briefly, the above is an outline of what is contemplated by the committee. A bill will be carefully drawn in due time for presentation to the Legislature and will be published in the medical journals of the State. The JOURNAL feels confident that we will this time secure the much needed legislation. The committee consists of Daniel and Tabor, of Austin; Paschal, of San Antonio; Saunders, of Fort Worth; Coleman, of Colorado, and Blaylock, of McGregor.

